# Red Cell Distribution Width-Standard Deviation Is Associated with Cumulative Metabolic Burden but Not Independently with Metabolic Syndrome

**DOI:** 10.3390/medicina62040647

**Published:** 2026-03-28

**Authors:** Kemal Ozan Lule, Nezihe Otay Lule, Mert Deniz Savcilioglu, Hamit Yildiz

**Affiliations:** 1 Internal Medicine Department, Faculty of Medicine, Gaziantep University, 27310 Gaziantep, Türkiye; drhyildiz@hotmail.com; 2 Nutrition and Dietetics Department, Faculty of Health Sciences, Gaziantep University, 27310 Gaziantep, Türkiye; otaynezihe@hotmail.com; 3 Department of Cardiology, Faculty of Medicine, Gaziantep University, 27310 Gaziantep, Türkiye; mdsavcilioglu@gmail.com

**Keywords:** metabolic syndrome, red cell distribution width, RDW-SD, cardiometabolic risk, inflammation, metabolic burden

## Abstract

*Background and Objectives*: Red cell distribution width (RDW) has been associated with adverse cardiometabolic outcomes; however, whether RDW—particularly RDW standard deviation (RDW-SD)—represents an independent determinant of metabolic syndrome (MetS) or reflects cumulative metabolic burden remains unclear. This study evaluated the association between RDW-SD and MetS presence and examined its relationship with the quantitative accumulation of MetS components. *Materials and Methods*: In this single-center observational study, 222 adults undergoing evaluation for MetS were consecutively recruited. Participants with overt anemia, extreme mean corpuscular volume values, or acute inflammation were excluded. MetS was defined according to revised NCEP ATP-III criteria. Associations between RDW-SD and MetS were assessed using hierarchical multivariable logistic regression models. The relationship between RDW-SD and the number of MetS components was examined using multivariable linear regression. Discriminative performance was evaluated by receiver operating characteristic (ROC) curve analysis. *Results*: MetS was present in 68.0% of participants. RDW-SD levels were significantly higher in individuals with MetS and increased progressively across quartiles. RDW-SD was independently associated with the number of MetS components (standardized β = 0.226, *p* < 0.001). However, RDW-SD was not independently associated with MetS presence in fully adjusted logistic models (OR = 1.07, 95% CI: 0.97–1.18, *p* = 0.198). The addition of RDW-SD provided minimal incremental explanatory value (Nagelkerke R^2^ increase from 0.348 to 0.356). ROC analysis demonstrated poor discriminatory ability (area under the curve [AUC] = 0.611, 95% CI: 0.535–0.687), supporting limited standalone diagnostic utility. *Conclusions*: RDW-SD was independently associated with cumulative metabolic burden but not with the independent presence of MetS after adjustment for established cardiometabolic factors. Given the cross-sectional design, these findings should be interpreted as associative rather than causal.

## 1. Introduction

Metabolic syndrome (MetS) represents a clustering of central obesity, dysglycemia, hypertension, and atherogenic dyslipidemia and is strongly associated with the development of type 2 diabetes mellitus, cardiovascular disease, and premature mortality [1,2]. Globally, the prevalence of MetS has been reported to range from 12.5% to 31.4%, depending on the diagnostic definition applied, underscoring its substantial and growing public health burden [3]. Metabolic syndrome reflects a state of persistent metabolic and inflammatory burden rather than a simple aggregation of risk factors, with chronic low-grade inflammation and oxidative stress playing central roles in adipose and endothelial dysfunction [4,5]. These interrelated mechanisms promote insulin resistance and vascular injury, while also influencing hematopoietic regulation and red blood cell (RBC) homeostasis.

Red cell distribution width (RDW), routinely reported as part of the complete blood count, quantifies variability in erythrocyte size (anisocytosis). Although traditionally used in the differential diagnosis of anemia, RDW has increasingly been recognized as a marker associated with cardiovascular morbidity, metabolic disorders, and all-cause mortality [6,7]. Experimental and clinical data suggest that inflammatory cytokines, oxidative stress, and impaired iron metabolism may disrupt erythropoiesis, shorten RBC lifespan, and increase heterogeneity in erythrocyte volume, thereby elevating RDW values [7,8]. RDW may be reported using two related but distinct indices: RDW-coefficient of variation (RDW-CV) and RDW-standard deviation (RDW-SD). RDW-CV reflects erythrocyte size variability relative to mean corpuscular volume, whereas RDW-SD represents the absolute width of the red cell volume distribution and is less dependent on mean corpuscular volume. For this reason, RDW-SD may provide a more direct and stable representation of anisocytosis in metabolically heterogeneous populations [9,10].

Several recent studies have examined associations between RDW and cardiometabolic risk factors; however, most have focused on RDW-CV and evaluated MetS as a dichotomous entity (present vs. absent) [11,12]. Considering MetS solely as a dichotomous condition may fail to capture the incremental metabolic and inflammatory burden reflected by its individual components. Accordingly, recent studies have increasingly used component count–based or cumulative metabolic burden approaches to better reflect graded metabolic stress beyond binary syndrome classification [13,14,15,16]. Quantifying the number of MetS components may therefore provide a more nuanced representation of this cumulative biological stress. Data exploring dose–response relationships between RDW—particularly RDW-SD—and quantitative MetS component count remain limited.

Furthermore, it remains unclear whether elevated RDW represents an independent determinant of MetS or rather reflects concomitant metabolic and inflammatory disturbances. Clarifying this relationship may help determine whether RDW-SD represents a biologically meaningful correlate of metabolic load or merely parallels established cardiometabolic risk factors.

However, it remains insufficiently understood whether RDW-SD is more closely associated with the cumulative clustering of MetS components than with the independent binary presence of MetS after multivariable adjustment, particularly in studies that distinguish RDW-SD from RDW-CV. Therefore, the present study had three aims: (1) to compare RDW-SD levels according to MetS status; (2) to evaluate the association between RDW-SD and the number of MetS components as an index of cumulative metabolic burden; and (3) to examine whether RDW-SD remained independently associated with MetS after adjustment for demographic, clinical, and inflammatory covariates.

## 2. Materials and Methods

### 2.1. Study Design and Population

This cross-sectional observational study included adults aged 18 to 65 years who underwent evaluation for metabolic syndrome during routine outpatient assessment. Adults who met the prespecified eligibility criteria were prospectively and consecutively recruited between 4 February and 20 February 2026, and all clinical and laboratory variables were assessed at a single time point for each participant. Eligible participants were those who provided written informed consent and had complete clinical and laboratory data required for metabolic syndrome classification and hematologic analysis, including waist circumference, blood pressure, fasting plasma glucose, triglycerides, HDL-cholesterol (HDL-C), and complete blood count parameters. A priori sample size calculation was performed based on a similar previously published cross-sectional study [17]. With α = 0.05 and 1−β = 0.80, the minimum required sample size was calculated as 109 participants using G*Power 3.9.1. The final sample included 222 eligible participants.

Participants were excluded if they had overt anemia (hemoglobin < 12 g/dL in women and <13 g/dL in men), mean corpuscular volume (MCV) < 80 fL or >100 fL, pregnancy, known hematologic disorders (such as hemolytic anemia or hematologic malignancy), evidence of acute infection or acute inflammatory status (C-reactive protein [CRP] > 10 mg/L), recent surgery within the previous 3 months, known liver disease, elevated serum creatinine levels suggestive of clinically relevant renal dysfunction, or missing clinical or laboratory data required for the analysis.

The study protocol was approved by the Gaziantep University Non-Interventional Clinical Research Ethics Committee (decision no: 2026/100, approval date: 4 February 2026). Written informed consent was obtained from all participants. The study was conducted in accordance with the Declaration of Helsinki. This study was reported in accordance with the Strengthening the Reporting of Observational Studies in Epidemiology (STROBE) guidelines.

### 2.2. Clinical and Anthropometric Assessment

Age, sex, smoking status, comorbidities, and medication use were recorded. Smoking status was categorized as never, former, or current smoker. Body weight and height were measured using standardized procedures, and body mass index (BMI) was calculated as kg/m^2^. Waist circumference was measured at the midpoint between the lowest rib and the iliac crest at the end of expiration. All anthropometric and blood pressure measurements were obtained by trained outpatient clinic personnel using standardized clinical procedures. Blood pressure was measured after at least 5 min of rest in the seated position, and the average of two measurements was recorded.

### 2.3. Laboratory Measurements

Venous blood samples were collected after a 12 h overnight fast. Complete blood count parameters, including RDW-SD, hemoglobin, hematocrit, mean corpuscular volume, and white blood cell count, were measured using an automated hematology analyzer (Sysmex XN-9000, Sysmex Corporation, Kobe, Japan) in the central laboratory of a tertiary-care university hospital under standardized operating conditions and routine internal quality control procedures. The laboratory reference range for RDW-SD in our institution was 36.3–47.3 fL. Fasting plasma glucose, triglycerides (TG), HDL-C, LDL-cholesterol [LDL-C], HbA1c, and CRP levels were analyzed using standard laboratory methods.

### 2.4. Definition of Metabolic Syndrome

Metabolic syndrome was defined in accordance with the revised National Cholesterol Education Program Adult Treatment Panel III (NCEP ATP-III) criteria [18]. Although country-specific waist circumference thresholds have been proposed for the Turkish population, these values have not been fully standardized across studies. Therefore, NCEP ATP-III–based waist circumference cutoffs were retained to ensure methodological consistency and comparability with the international literature. A diagnosis of MetS required the presence of at least three of the following five components:

• Abdominal obesity (waist circumference ≥ 102 cm in men or ≥88 cm in women);
• Elevated fasting plasma glucose (≥100 mg/dL or antidiabetic treatment);
• Elevated triglycerides (≥150 mg/dL or lipid-lowering treatment);
• Reduced HDL-C (<40 mg/dL in men or <50 mg/dL in women, or drug treatment for reduced HDL-C);
• Elevated blood pressure (≥130/85 mmHg or antihypertensive treatment).

The number of MetS components was calculated for each participant (range: 0–5) to represent cumulative metabolic burden. This variable was considered an ordered summary index of cumulative metabolic burden and was modeled as an approximately continuous variable in linear regression analyses for interpretability.

### 2.5. Statistical Analysis

All statistical analyses were conducted using IBM SPSS Statistics version 22.0 (IBM Corp., Armonk, NY, USA). Continuous variables were assessed for normality using visual inspection (histograms and Q-Q plots) and the Shapiro–Wilk test. Normally distributed variables are presented as mean ± standard deviation, whereas non-normally distributed variables are reported as median (interquartile range). Categorical variables are expressed as number (percentage).

Between-group comparisons (MetS present vs. absent) were performed using the independent samples *t*-test or Mann–Whitney U test for continuous variables, as appropriate. Categorical variables were compared using the chi-square test. To evaluate the relationship between RDW-SD and metabolic burden, correlation analyses were conducted using Pearson or Spearman coefficients according to distribution characteristics. In addition, multivariable linear regression analysis was performed to assess the independent association between RDW-SD and the number of MetS components, adjusting for demographic and clinical covariates.

To determine independent predictors of MetS presence, hierarchical multivariable logistic regression analysis was performed using sequentially adjusted models. Variables included in the main multivariable models were selected a priori based on clinical relevance and to avoid overadjustment with factors directly embedded in the MetS definition. CRP was included as a non-criterion inflammatory covariate, whereas HDL-C and HbA1c were not included in the primary logistic models because HDL-C is part of the MetS definition and HbA1c substantially overlaps with the glycemic component of the syndrome. Smoking status was categorized as never, former, or current smoker. For regression analyses, smoking was entered as two dummy variables (former and current smoking), with never smokers serving as the reference category.

• In Model 1 (demographic model), age, sex, and smoking status were included.
• In Model 2 (clinical-adjusted model), BMI and CRP were added to Model 1.
• In Model 3 (fully adjusted model), RDW-SD was entered to evaluate its incremental contribution beyond established demographic and clinical risk factors.

Model fit was assessed using the Hosmer–Lemeshow goodness-of-fit test, and explained variance was reported using Cox–Snell and Nagelkerke R^2^ statistics. Multicollinearity was evaluated using variance inflation factor (VIF) and tolerance values. Receiver operating characteristic (ROC) curve analysis was performed to evaluate the discriminative performance of RDW-SD for MetS presence, and the area under the curve (AUC) was calculated. Additionally, participants were categorized into quartiles according to RDW-SD levels, and trends in MetS prevalence across quartiles were assessed using the chi-square test and linear-by-linear association analysis. To assess the robustness of the findings, sensitivity analyses were conducted by additionally adjusting the main regression model for hemoglobin (HGB) and HbA1c separately (Supplementary Table S1). An additional sensitivity logistic regression analysis excluding BMI was also performed to evaluate whether adiposity adjustment attenuated the association between RDW-SD and MetS (Supplementary Table S2). A two-sided *p*-value < 0.05 was considered statistically significant.

## 3. Results

### 3.1. Baseline Characteristics of the Study Population

Baseline characteristics of the study population according to metabolic syndrome (MetS) status are presented in Table 1. Among 222 participants, 151 (68.0%) were classified as having MetS. Participants with MetS were significantly older than those without MetS (52.94 ± 7.84 vs. 48.52 ± 11.82 years, *p* < 0.05). The prevalence of female sex and current smoking was higher in the MetS group (68.2% vs. 46.5% and 66.2% vs. 47.9%, respectively; both *p* < 0.05). Body mass index (BMI) was markedly higher among individuals with MetS (31.69 ± 4.50 vs. 27.36 ± 4.48 kg/m^2^, *p* < 0.001). As expected, the mean number of MetS components was significantly greater in the MetS group (3.72 ± 0.72 vs. 1.62 ± 0.62, *p* < 0.001).

Red cell distribution width–standard deviation (RDW-SD) levels were significantly elevated in participants with MetS compared to those without MetS (43.74 ± 3.83 vs. 42.02 ± 3.23 fL, *p* < 0.05). Hemoglobin and mean corpuscular volume (MCV) were modestly but significantly lower in the MetS group (both *p* < 0.05), whereas hematocrit showed a borderline difference (*p* = 0.053).

Inflammatory and metabolic parameters differed significantly between groups. C-reactive protein (CRP), glycated hemoglobin (HbA1c), fasting plasma glucose, and TG levels were higher in individuals with MetS (all *p* < 0.05), while HDL-C levels were significantly lower (*p* < 0.001). No statistically significant differences were observed in LDL-C or white blood cell (WBC) count (*p* > 0.05). Platelet count (PLT) was modestly higher in the MetS group (*p* < 0.05)

### 3.2. Correlation of RDW-SD with MetS Component Count

The number of MetS components showed significant positive correlations with age, BMI, and waist circumference, and significant negative correlations with hemoglobin (HGB), hematocrit (HCT), mean corpuscular volume (MCV), and HDL-C (all *p* < 0.05). In addition, component count was positively correlated with WBC, CRP, HbA1c, fasting plasma glucose, and triglyceride levels (all *p* < 0.05), whereas no significant association was observed with LDL-C (*p* > 0.05).

RDW-SD levels demonstrated significant positive correlations with age, BMI, and waist circumference, and significant negative correlations with HGB and HDL-C (all *p* < 0.05). Furthermore, RDW-SD was positively associated with WBC and CRP levels (*p* < 0.05). No significant correlations were observed between RDW-SD and HCT, MCV, HbA1c, LDL-C, platelet count, fasting plasma glucose, or triglyceride levels (all *p* > 0.05) (Table 2).

### 3.3. RDW-SD According to Individual MetS Components

When RDW-SD levels were evaluated according to individual MetS components, significantly higher RDW-SD values were observed in participants with hypertension, reduced HDL-C levels, abdominal obesity, and elevated fasting plasma glucose (all *p* < 0.05). Among these components, the largest effect sizes were observed for abdominal obesity (Cohen’s d = 0.56) and hypertension (Cohen’s d = 0.51). A comparable effect size was noted for reduced HDL-C (Cohen’s d = 0.49), whereas the effect size for elevated fasting plasma glucose was smaller (Cohen’s d = 0.34). In contrast, no statistically significant difference in RDW-SD levels was observed between participants with and without hypertriglyceridemia (*p* = 0.406), and the corresponding effect size was negligible (Cohen’s d = 0.11) (Table 3).

### 3.4. MetS Prevalence Across RDW-SD Quartiles

When participants were stratified according to RDW-SD quartiles, the prevalence of MetS was overall higher in the upper quartiles and reached its highest level in Q4. MetS prevalence was 59.3% in the lowest quartile (Q1) and 85.7% in the highest quartile (Q4). The overall distribution of MetS across quartiles was statistically significant (chi-square *p* = 0.010), and linear trend analysis also showed a significant positive trend across increasing RDW-SD quartiles (*p* for trend = 0.008) (Table 4).

### 3.5. Multivariable Linear Regression for MetS Component Count

Multivariable linear regression analysis was performed to evaluate independent determinants of the number of MetS components (Table 5). RDW-SD was independently and positively associated with MetS component count (standardized β = 0.226, *p* < 0.001). Age (β = 0.178, *p* = 0.002), female sex (β = 0.199, *p* = 0.002), current smoking (β = 0.199, *p* = 0.002), and BMI (β = 0.291, *p* < 0.001) were also independently associated with a higher number of MetS components. CRP and former smoking were not significantly associated with component count. The overall model was statistically significant (F = 15.11, *p* < 0.001) and explained 33.1% of the variance in MetS component count (R^2^ = 0.331; adjusted R^2^ = 0.309). Multicollinearity was not observed (all VIF values <2.0).

### 3.6. Hierarchical Logistic Regression for MetS Presence

Hierarchical multivariable logistic regression analysis was performed to evaluate independent predictors of MetS across sequentially adjusted models (Table 6). In Model 1, female sex and current smoking were significantly associated with MetS, and age demonstrated a significant positive association. This model explained 19.9% of the variance (Nagelkerke R^2^ = 0.199). After adding clinical variables in Model 2, BMI emerged as one of the strongest independent predictors of MetS, while CRP was not independently associated. The inclusion of clinical variables substantially improved model explanatory power (Nagelkerke R^2^ = 0.348).

In Model 3 (fully adjusted model), RDW-SD was added to assess its incremental contribution. RDW-SD was not independently associated with MetS (OR = 1.07, 95% CI: 0.97–1.18, *p* = 0.198). The addition of RDW-SD resulted in only a minimal increase in model explanatory capacity (Nagelkerke R^2^ = 0.356).

In the final model, female sex, current smoking, and BMI remained independently associated with MetS. Model calibration was adequate (Hosmer-Lemeshow *p* = 0.320), and the overall classification accuracy was 78.4%. No significant multicollinearity was observed (all VIF values < 2.0).

### 3.7. Sensitivity and ROC Analyses

To assess the robustness of the main findings, additional sensitivity analyses were conducted by incorporating hemoglobin and HbA1c into the final model. The inclusion of hemoglobin did not materially change the associations observed in the primary model, and RDW-SD remained non-significant. Similarly, after adjustment for HbA1c, RDW-SD was not independently associated with MetS, whereas HbA1c emerged as a significant predictor. Across both sensitivity models, BMI and active smoking consistently remained independently associated with MetS (Supplementary Table S1). In an additional sensitivity logistic regression analysis excluding BMI, RDW-SD became significantly associated with MetS presence (B = 0.109, OR = 1.115, 95% CI: 1.013–1.227, *p* = 0.026). The BMI-excluded model showed acceptable fit (Nagelkerke R^2^ = 0.245, Hosmer–Lemeshow *p* = 0.939) and an overall classification accuracy of 76.1% (Supplementary Table S2).

**Figure 1:**
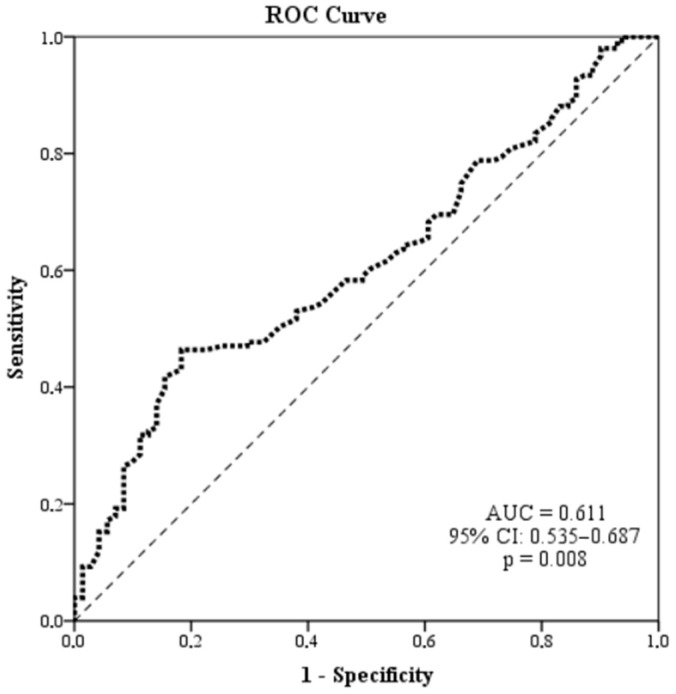
Receiver operating characteristic (ROC) curve of RDW-SD for discriminating the presence of metabolic syndrome. The dashed diagonal line indicates the reference line of no discrimination.

ROC analysis showed that RDW-SD had poor discriminatory performance for MetS (AUC = 0.611, 95% CI: 0.535–0.687, *p* = 0.008). Using the Youden index, the optimal cutoff value was 43.90 fL, yielding a sensitivity of 46.4%, specificity of 81.7%, positive predictive value of 84.4%, and negative predictive value of 41.7% (Figure 1).

## 4. Discussion

The present study suggests that RDW-SD is independently associated with cumulative metabolic burden, as reflected by the number of MetS components, but does not independently predict the presence of MetS after adjustment for established cardiometabolic risk factors. Although RDW-SD levels increased progressively across quartiles and were moderately correlated with component count, its discriminative performance for identifying MetS was limited (AUC = 0.611). Dichotomization of MetS may result in information loss and attenuate gradient effects that are more readily detectable in continuous component-based models, potentially explaining the discrepancy between component count analyses and binary MetS classification. Taken together, these findings support RDW-SD as a correlate of metabolic load rather than a standalone diagnostic marker.

Recent literature increasingly conceptualizes MetS as a systemic pro-inflammatory and pro-oxidative condition extending beyond a simple clustering of risk factors [19]. Chronic low-grade inflammation, endothelial dysfunction, and adipose tissue-derived cytokine signaling are now considered central drivers of metabolic dysregulation [20]. Within this framework, alterations in erythrocyte morphology—captured by RDW indices—may represent a biological imprint of sustained inflammatory and metabolic stress.

Multiple large-scale population-based analyses have demonstrated that elevated RDW is associated with increased mortality and adverse cardiometabolic outcomes [21,22]. Higher RDW levels have been independently associated with all-cause and cardiovascular mortality, supporting its role as an integrative marker of systemic vulnerability [21]. Similarly, elevated RDW has been shown to predict cardiovascular mortality across ASCVD risk cohorts [22]. However, these investigations primarily evaluated hard clinical outcomes and relied predominantly on RDW-CV. They did not specifically examine RDW-SD in relation to the incremental burden of metabolic syndrome components or apply hierarchical modeling approaches to distinguish whether RDW-SD is more closely associated with binary MetS status or with the graded accumulation of MetS components.

While RDW has been consistently associated with adverse cardiometabolic outcomes and mortality [21,22], our findings suggest that, within the metabolic syndrome phenotype, RDW-SD is more closely linked to cumulative metabolic clustering than to the dichotomous presence of MetS after multivariable adjustment. Prior evidence has also linked RDW and other hematologic parameters to metabolic syndrome and related abnormalities [23]. Accordingly, RDW-SD may be better interpreted as a correlate of underlying metabolic and inflammatory burden rather than an independent determinant of MetS. However, given the cross-sectional design, these interpretations should remain associative and hypothesis-generating rather than causal or confirmatory.

From a mechanistic standpoint, chronic low-grade inflammation and oxidative stress may impair erythropoiesis, disrupt iron homeostasis, and shorten red blood cell lifespan, thereby increasing anisocytosis [24,25]. Oxidative stress, in particular, can alter red blood cell membrane integrity and deformability through reactive oxygen species–mediated damage, potentially accelerating erythrocyte turnover and structural heterogeneity [24]. These processes provide a plausible biological explanation for elevated RDW-SD in the setting of sustained metabolic and inflammatory stress.

A methodological strength of our study is the focus on RDW-SD rather than RDW-CV. As RDW-SD reflects absolute variability in erythrocyte volume and is less dependent on mean corpuscular volume [26], it may provide a more stable representation of anisocytosis in metabolically heterogeneous populations. Our findings are also broadly consistent with prior meta-analytic evidence linking RDW parameters to metabolic syndrome [27], although most previous studies evaluated syndrome presence rather than component accumulation.

Notably, among the individual MetS components, the strongest association with RDW-SD was observed for abdominal obesity. This finding may be biologically plausible, as central adiposity is more closely linked than overall adiposity to chronic low-grade inflammation, adipokine dysregulation, endothelial dysfunction, and oxidative stress, all of which may adversely affect erythropoiesis and erythrocyte size heterogeneity. Prior evidence has also suggested that the relationship between RDW and MetS may be particularly influenced by abdominal obesity, supporting the view that visceral adiposity may be an important contributor to the hematologic alterations captured by RDW indices [28]. In this context, the stronger effect size observed for abdominal obesity in our study may indicate that RDW-SD is more sensitive to central metabolic and inflammatory burden than to some other individual MetS components.

In an additional sensitivity analysis excluding BMI, RDW-SD became significantly associated with MetS presence. This finding suggests that adjustment for adiposity may have attenuated the association observed in the primary model. Given the central role of adiposity in the pathophysiological complexity of metabolic syndrome, such attenuation is biologically plausible [29]. Nevertheless, this finding does not support interpreting RDW-SD as an adiposity-independent determinant of MetS; rather, it indicates that the association is context-dependent and influenced by the broader metabolic milieu.

Clinically, our findings do not support the use of RDW-SD as a standalone screening or diagnostic tool for MetS. However, given its universal availability within routine complete blood count testing, RDW-SD may still provide adjunctive insight into cumulative metabolic and inflammatory burden. Prospective longitudinal studies are needed to determine whether RDW-SD offers incremental prognostic information for incident MetS or broader cardiometabolic outcomes beyond established risk factors.

### Limitations

Several limitations should be acknowledged. First, although participants were prospectively recruited, the observational cross-sectional nature of the analysis precludes causal inference. The temporal direction between RDW-SD and metabolic burden therefore cannot be definitively established, and interpretations regarding biological direction, mediation, or downstream effects should be considered hypothesis-generating. Second, despite careful exclusion of overt anemia, extreme MCV values (<80 fL or >100 fL), acute inflammatory status, and known liver disease, residual confounding cannot be entirely excluded. Iron status markers, vitamin B12, folate, and erythropoietic indices were not systematically available for all participants. More detailed assessment of subclinical renal, hepatic, nutritional, or inflammatory conditions was also unavailable in the full study sample. Therefore, unmeasured hematologic, nutritional, and metabolic factors may still have influenced RDW-SD levels. Third, RDW-SD was assessed at a single time point, and serial measurements were not available, limiting evaluation of longitudinal variability and prognostic significance. Fourth, the modest discriminative performance observed in ROC analyses indicates limited utility for individual-level screening or diagnostic purposes; thus, RDW-SD should be interpreted within a pathophysiological rather than diagnostic framework. Fifth, the specific hematology analyzer model was not available in the study dataset, although all complete blood count measurements were performed in the institutional central laboratory under routine internal quality control procedures. Finally, the relatively high prevalence of MetS in this outpatient sample likely reflects the clinical setting of recruitment rather than population prevalence, and together with the single-center design, may limit generalizability to broader community-based populations and to populations with different demographic or metabolic characteristics.

## 5. Conclusions

In conclusion, RDW-SD was not independently associated with MetS after adjustment for established metabolic and hematologic covariates, although it remained associated with the cumulative number of MetS components. These findings suggest that RDW-SD is more closely linked to overall metabolic clustering than to the dichotomous presence of MetS. Because of the cross-sectional design, the observed relationships should be interpreted as associations rather than evidence of causal or temporal directionality. While RDW-SD lacks standalone diagnostic utility, its routine availability within complete blood count testing may offer adjunctive insight into systemic metabolic burden. Prospective studies are warranted to determine its longitudinal prognostic relevance.

## Figures and Tables

**Table 1:** Participant characteristics according to MetS status.

Variable	Total (*n* = 222)	No MetS (*n* = 71)	MetS (*n* = 151)	*p*-Value
Age (years)	51.53 ± 9.50	48.52 ± 11.82	52.94 ± 7.84	**<0.05**
Female sex, *n* (%)	136 (61.3)	33 (46.5)	103 (68.2)	**<0.05**
Current smoking, *n* (%)	134 (60.4)	34 (47.9)	100 (66.2)	**<0.05**
BMI (kg/m^2^)	30.31 ± 4.92	27.36 ± 4.48	31.69 ± 4.50	**<0.001**
Number of MetS components	3.05 ± 1.20	1.62 ± 0.62	3.72 ± 0.72	**<0.001**
RDW-SD (fL)	43.19 ± 3.73	42.02 ± 3.23	43.74 ± 3.83	**<0.05**
Hemoglobin (g/dL)	14.39 ± 1.36	14.77 ± 1.43	14.22 ± 1.29	**<0.05**
Hematocrit (%)	43.35 ± 3.61	44.03 ± 3.82	43.02 ± 3.47	0.053
MCV (fL)	86.82 ± 4.01	88.05 ± 3.88	86.24 ± 3.95	**<0.05**
WBC (×10^3^/µL)	8.51 ± 2.02	8.43 ± 2.24	8.55 ± 1.92	0.692
CRP (mg/L)	4.21 ± 2.61	3.49 ± 2.56	4.54 ± 2.57	**<0.05**
Fasting glucose (mg/dL), median (IQR)	132 (97–192)	98 (87–154)	149 (108–197)	**<0.001**
HbA1c (%)	7.60 ± 2.23	6.87 ± 2.28	7.93 ± 2.13	**<0.05**
TG (mg/dL), median (IQR)	171 (123–237.5)	125 (103–149)	187 (148–268)	**<0.001**
HDL-C (mg/dL)	49.80 ± 12.34	55.01 ± 13.82	47.34 ± 10.79	**<0.001**
LDL-C (mg/dL)	130.75 ± 30.78	130.30 ± 31.32	130.97 ± 30.63	0.880
PLT (×10^3^/µL)	286.35 ± 71.25	268.41 ± 60.10	294.79 ± 74.64	**<0.05**

Data are presented as mean ± standard deviation or median (interquartile range), according to distribution. Between-group comparisons were performed using independent samples *t*-test or Mann–Whitney U test for continuous variables and chi-square test for categorical variables. Statistically significant *p*-values are shown in bold. Abbreviations: MetS, metabolic syndrome; BMI, body mass index; RDW-SD, red cell distribution width–standard deviation; HGB, hemoglobin; HCT, hematocrit; MCV, mean corpuscular volume; WBC, white blood cell count; CRP, C-reactive protein; HbA1c, glycated hemoglobin; TG, triglycerides; HDL-C, high-density lipoprotein cholesterol; LDL-C, low-density lipoprotein cholesterol; PLT, platelet count; IQR, interquartile range.

**Table 2:** Correlations Between MetS Component Count, RDW-SD, and Clinical and Laboratory Parameters.

Variable	MetS Component Count (r)	RDW-SD (r)
Age	0.281 **	0.140 *
BMI	0.425 **	0.228 **
Waist circumference	0.512 **	0.224 **
HGB	−0.232 **	−0.206 **
HCT	−0.182 **	−0.108
MCV	−0.256 **	0.064
WBC	0.132 *	0.246 **
CRP	0.151 *	0.148 *
Fasting plasma glucose ^†^	0.342 **	−0.050
HbA1c	0.231 **	0.026
TG ^†^	0.477 **	0.006
HDL-C	−0.424 **	−0.183 **
LDL-C	0.015	−0.065
PLT	0.149 *	0.107
RDW-SD	0.346 **	-

Pearson correlation coefficients were used unless otherwise specified. Spearman correlation coefficients were used for fasting plasma glucose and triglycerides (^†^). * *p* < 0.05, ** *p* < 0.01. Abbreviations: MetS, metabolic syndrome; RDW-SD, red cell distribution width–standard deviation; BMI, body mass index; HGB, hemoglobin; HCT, hematocrit; MCV, mean corpuscular volume; WBC, white blood cell count; CRP, C-reactive protein; HbA1c, glycated hemoglobin; HDL-C, high-density lipoprotein; LDL-C, low-density lipoprotein; TG, triglycerides; PLT, platelet count.

**Table 3:** RDW-SD Levels and Effect Sizes According to Individual MetS Components.

Component	Absent (Mean ± SD)	Present (Mean ± SD)	*p*-Value	Cohen’s d
Hypertension	42.21 ± 3.44	44.02 ± 3.77	**<0.001**	0.51
Reduced HDL-C	42.51 ± 3.16	44.29 ± 4.29	**<0.05**	0.49
Abdominal obesity	41.56 ± 3.57	43.60 ± 3.66	**<0.05**	0.56
Elevated fasting plasma glucose	42.26 ± 2.96	43.51 ± 3.91	**<0.05**	0.34
Elevated TG	42.94 ± 3.41	43.36 ± 3.94	0.406	0.11

Data are presented as mean ± standard deviation. Between-group comparisons were performed using independent samples *t*-test. Effect size was calculated using Cohen’s d. Statistically significant *p*-values are shown in bold. Abbreviations: RDW-SD, red cell distribution width–standard deviation; HDL-C, high-density lipoprotein; TG, triglycerides.

**Table 4:** Prevalence of Metabolic Syndrome According to RDW-SD Quartiles.

RDW-SD Quartile	MetS Absent *n* (%)	MetS Present *n* (%)
Q1	22 (40.7)	32 (59.3)
Q2	19 (33.9)	37 (66.1)
Q3	22 (39.3)	34 (60.7)
Q4	8 (14.3)	48 (85.7)

Chi-square *p* = 0.010, *p* for trend = 0.008.

**Table 5:** Multivariable Linear Regression Analysis for MetS Component Count.

Variable	B	Standardized β	*p*-Value
RDW-SD	0.073	0.226	**<0.001**
Age	0.022	0.178	**0.002**
Female sex	0.490	0.199	**0.002**
Current smoking	0.487	0.199	**0.002**
Former smoking	0.165	0.024	0.678
BMI	0.071	0.291	**<0.001**
CRP	0.000	−0.001	0.990

Model performance: R^2^ = 0.331, Adjusted R^2^ = 0.309, F = 15.11, *p* < 0.001; all VIF values were <2.0. Statistically significant *p*-values are shown in bold.

**Table 6:** Hierarchical Logistic Regression Analysis for MetS Presence.

Variable	Model 1 B	Model 1 OR (95% CI)	*p*	Model 2 B	Model 2 OR (95% CI)	*p*	Model 3 B	Model 3 OR (95% CI)	*p*
Age	0.043	1.04 (1.01–1.08)	**0.007**	0.036	1.04 (1.00–1.07)	**0.041**	0.033	1.03 (0.99–1.07)	0.066
Female sex	1.535	4.64 (2.24–9.64)	**<0.001**	1.186	3.27 (1.49–7.17)	**0.003**	1.205	3.34 (1.52–7.34)	**0.003**
Former smoking	0.214	1.24 (0.24–6.50)	0.800	0.391	1.48 (0.28–7.69)	0.642	0.276	1.32 (0.24–7.25)	0.751
Current smoking	1.333	3.79 (1.79–8.03)	**<0.001**	1.180	3.25 (1.45–7.32)	**0.004**	1.154	3.17 (1.41–7.16)	**0.005**
BMI	—	—	—	0.202	1.22 (1.12–1.34)	**<0.001**	0.192	1.21 (1.11–1.32)	**<0.001**
CRP	—	—	—	0.050	1.05 (0.92–1.20)	0.460	0.042	1.04 (0.91–1.19)	0.545
RDW-SD	—	—	—	—	—	—	0.065	1.07 (0.97–1.18)	0.198

Model 1: age, sex, smoking, Model 2: Model 1 + BMI + CRP, Model 3: Model 2 + RDW-SD. (Model fit: Model 1 Nagelkerke R^2^ = 0.199, Model 2 Nagelkerke R^2^ = 0.348, Model 3 Nagelkerke R^2^ = 0.356, Hosmer–Lemeshow *p* = 0.320, Overall classification accuracy = 78.4%, All VIF values were <2.0). Abbreviations: MetS, metabolic syndrome; BMI, body mass index; CRP, C-reactive protein; RDW-SD, red cell distribution width-standard deviation; CI, confidence interval; VIF, variance inflation factor. Statistically significant *p*-values are shown in bold.

## Data Availability

The datasets generated and/or analyzed during the current study are not publicly available due to institutional data protection policies but are available from the corresponding author on reasonable request.

## Supplementary Materials

The following supporting information can be downloaded at: https://www.mdpi.com/article/10.3390/medicina62040647/s1, Table S1: Sensitivity Analyses for the Association Between RDW-SD and Metabolic Syndrome; Table S2: Hierarchical Logistic Regression Sensitivity Analysis for Metabolic Syndrome Presence Excluding BMI.

## Abbreviations

The following abbreviations are used in this manuscript:

AUC	Area Under the Curve
BMI	Body Mass Index
CI	Confidence Interval
CRP	C-Reactive Protein
HDL-C	High-Density Lipoprotein
HCT	Hematocrit
HGB	Hemoglobin
HbA1c	Glycated Hemoglobin
IQR	Interquartile Range
LDL-C	Low-Density Lipoprotein
MCV	Mean Corpuscular Volume
MetS	Metabolic Syndrome
NCEP ATP-III	National Cholesterol Education Program Adult Treatment Panel III
OR	Odds Ratio
PLT	Platelet Count
R^2^	Coefficient of Determination
RBC	Red Blood Cell
RDW	Red Cell Distribution Width
RDW-CV	Red Cell Distribution Width–Coefficient of Variation
RDW-SD	Red Cell Distribution Width–Standard Deviation
ROC	Receiver Operating Characteristic
TG	Triglycerides
VIF	Variance Inflation Factor
WBC	White Blood Cell Count
