# Supplementary material for: Red Cell Distribution Width-Standard Deviation Is Associated with Cumulative Metabolic Burden but Not Independently with Metabolic Syndrome
Source: Medicina (Kaunas). 2026 Mar 28;62(4):647. doi: 10.3390/medicina62040647 (PMC13117053; doi:10.3390/medicina62040647)
Supplement: Supplementary file 1 [file medicina-62-00647-s001.zip › Supplementary Table S1.pdf]

Supplementary Table S1. Sensitivity analysis of the association between RDW-SD and metabolic syndrome using hemoglobin- and HbA1c-adjusted logistic regression models

**Variable**	**Hemoglobin-adjusted model OR (95% CI)**	**p-value**	**HbA1c-adjusted model OR (95% CI)**	**p-value**
Age	1.03 (0.99–1.07)	0.085	1.04 (1.00–1.08)	0.061
Female sex	2.24 (0.87–5.77)	0.095	3.81 (1.69–8.57)	**0.001**
Current smoking	4.05 (1.67–9.83)	**0.002**	3.04 (1.32–6.99)	**0.009**
BMI	1.21 (1.11–1.33)	**<0.001**	1.23 (1.12–1.35)	**<0.001**
CRP	1.04 (0.90–1.19)	0.608	1.01 (0.88–1.17)	0.881
RDW-SD	1.05 (0.94–1.16)	0.407	1.07 (0.97–1.19)	0.188
Hemoglobin	0.76 (0.53–1.10)	0.141	—	—
HbA1c	—	—	1.33 (1.13–1.57)	**0.001**

OR: Odds ratio, CI: Confidence interval, BMI: Body mass index, CRP: C-reactive protein, RDW-SD: Red cell distribution width–standard deviation, HbA1c: Glycated hemoglobin. Bold values indicate statistically significant associations ($p < 0.05$). Sensitivity analyses were performed to evaluate whether the association between RDW-SD and metabolic syndrome was influenced by hemoglobin levels or glycemic status. Two separate multivariable logistic regression models were constructed: one including hemoglobin and the other including HbA1c as additional covariates. Odds ratios represent the change in odds of metabolic syndrome per one-unit increase in continuous variables.
