# Supplementary material for: Red Cell Distribution Width-Standard Deviation Is Associated with Cumulative Metabolic Burden but Not Independently with Metabolic Syndrome
Source: Medicina (Kaunas). 2026 Mar 28;62(4):647. doi: 10.3390/medicina62040647 (PMC13117053; doi:10.3390/medicina62040647)
Supplement: Supplementary file 1 [file medicina-62-00647-s001.zip › Supplementary Table S2.pdf]

Supplementary Table S2. Sensitivity logistic regression analysis for MetS presence excluding BMI

| Variable | Model 1 B | Model 1 OR (95% CI) | p | Model 2 B | Model 2 OR (95% CI) | p | Mod el 3 B | Model 3 OR (95% CI) | p |
|---|---|---|---|---|---|---|---|---|---|
| Age | 0.043 | 1.04 (1.01–1.08) | **0.007** | 0.043 | 1.04 (1.01–1.08) | **0.007** | 0.037 | 1.04 (1.01–1.07) | **0.024** |
| Male sex (vs female) | -1.535 | 0.22 (0.10–0.45) | **<0.001** | -1.393 | 0.25 (0.12–0.52) | **<0.001** | -1.397 | 0.25 (0.12–0.53) | **<0.001** |
| Former smoking | 0.214 | 1.24 (0.24–6.50) | 0.800 | 0.170 | 1.19 (0.23–6.10) | 0.839 | 0.005 | 1.01 (0.18–5.66) | 0.995 |
| Current smoking | 1.333 | 3.79 (1.79–8.03) | **<0.001** | 1.241 | 3.46 (1.62–7.39) | **0.001** | 1.165 | 3.21 (1.49–6.90) | **0.003** |
| CRP | — | — | — | 0.115 | 1.12 (0.99–1.27) | 0.070 | 0.102 | 1.11 (0.98–1.26) | 0.113 |
| RDW-SD | — | — | — | — | — | — | 0.109 | 1.12 (1.01–1.23) | **0.026** |

OR: Odds ratio; CI: Confidence interval; BMI: Body mass index; CRP: C-reactive protein; RDW-SD: Red cell distribution width–standard deviation. Bold values indicate statistically significant associations ($p < 0.05$). A hierarchical logistic regression sensitivity analysis excluding BMI was performed to evaluate whether adiposity adjustment influenced the association between RDW-SD and metabolic syndrome. Model 1 included age, sex, and smoking status; Model 2 additionally included CRP; and Model 3 additionally included RDW-SD. Odds ratios represent the change in odds of metabolic syndrome per one-unit increase in continuous variables.
